# Chronic Stress Does Not Influence the Survival of Mouse Models of Glioblastoma

**DOI:** 10.3389/fonc.2022.856210

**Published:** 2022-03-25

**Authors:** Marta Lopes, Joana Vieira de Castro, Marta Pojo, Céline S. Gonçalves, Eduarda P. Martins, Bárbara Coimbra, Ioannis Sotiropoulos, Nuno Sousa, Ana João Rodrigues, Bruno M. Costa

**Affiliations:** ^1^ Life and Health Sciences Research Institute, School of Medicine, University of Minho, Braga, Portugal; ^2^ ICVS/3B’s – PT Government Associate Laboratory, Braga/Guimarães, Portugal

**Keywords:** glioblastoma, corticosterone, chronic stress, chronic restraint stress, chronic unpredictable stress, GL261, U87-MG, overall survival

## Abstract

The existence of a clear association between stress and cancer is still a matter of debate. Recent studies suggest that chronic stress is associated with some cancer types and may influence tumor initiation and patient prognosis, but its role in brain tumors is not known. Glioblastoma (GBM) is a highly malignant primary brain cancer, for which effective treatments do not exist. Understanding how chronic stress, or its effector hormones glucocorticoids (GCs), may modulate GBM aggressiveness is of great importance. To address this, we used both syngeneic and xenograft *in vivo* orthotopic mouse models of GBM, in immunocompetent C57BL/6J or immunodeficient NSG mice, respectively, to evaluate how different paradigms of stress exposure could influence GBM aggressiveness and animals’ overall survival (OS). Our results demonstrated that a previous exposure to exogenous corticosterone administration, chronic restraint stress, or chronic unpredictable stress do not impact the OS of these mice models of GBM. Concordantly, *ex vivo* analyses of various GBM-relevant genes showed similar intra-tumor expression levels across all experimental groups. These findings suggest that corticosterone and chronic stress do not significantly affect GBM aggressiveness in murine models.

## Introduction

The role of stress in cancer initiation and progression remains unclear, but it is known that stress can alter neuroendocrine and immune functions, along with having several implications in pathophysiological processes that are also fundamental to cancer growth and progression (1–3). Epidemiological studies have suggested that combination of chronic stress and low social support is associated to a nine-fold increase in breast cancer incidence (4). On the other hand, experimental animal studies have been providing evidence of the effects of stress on tumor progression. For instance, chronic restraint stress has been shown to promote colorectal tumor growth in nude mice *via* stimulation of colorectal carcinoma cell proliferation (1). Additionally, in a mouse model of breast cancer, chronic stress restructured the lymphatic networks within and around tumors to provide pathways for tumor cell dissemination (5). Similarly, several other studies have suggested that stress is associated with some types of cancers (e.g. pancreatic, prostate, ovarian, oral cancer), and may be a risk factor for cancer development and progression (1–3, 5–7). By contrast, it has been shown that the brain’s reward system can modulate an anti-tumor immune response in tumor-bearing mice (8). However, nothing is known on the putative role of stress in glioblastomas (GBMs).

GBMs are the most frequent and malignant primary brain tumors in adults (9, 10), being characterized by high levels of cellular proliferation, invasion, and necrotic regions, while presenting a remarkable inter- and intra-tumor heterogeneity (10, 11). Despite treatment advances, GBM remains among the top deadliest cancers with very poor prognosis (12–14). The median survival is approximately 15 months, and the 5-year survival rate of GBM patients is still less than 5% after diagnosis (15–18). Despite considerable progress in the understanding of the biological characteristics of GBM, their etiology has not been fully elucidated. Established risk factors only include exposure to high dose ionizing radiation that is believed to increase the likelihood of developing GBM (19). The full knowledge of the involvement of other risk factors that can have an impact in patient’s prognosis is of great importance towards more preventive measures in the future.

Stress is generally defined as an actual or anticipated threat of well-being or disruption of the organism homeostasis. The activation of the stress response is critical to improve an individual’s chance of survival, and to promote adaptation when facing threatful or aversive situations (20). Chronic stress is characterized by a maladaptive response to long-term exposure to stressors that initiate a cascade of reactions, including activation of the sympathetic nervous system (SNS) and the hypothalamic-pituitary-adrenal (HPA) axis (21). This leads to local and systemic elevated levels of catecholaminergic neurotransmitters and involves an endocrine response with an increased release of stress hormones, such as glucocorticoids (GCs) (20, 22). GCs execute a wide range of biological functions, including modulation of the immune, endocrine and inflammatory responses (23). Accumulating evidence supports the role of GCs signaling in the progression of cancer through increased cell proliferation, inhibition of apoptosis and impairment of DNA repair (24). GCs serum levels were associated with a reduction of patients survival in breast and lung cancers, as well as with acquired chemotherapy resistance through cell death impairment of tumor cells (23, 25, 26).

In this work, we studied the effects of elevated GCs and chronic stress levels in GBM aggressiveness and survival. For that, we used multiple orthotopic mouse models of GBM, including syngeneic mouse or xenografts human GBM cell lines, to determine the prognostic impact of GCs administration and chronic stress paradigms in GBM. This study provides the first insights into understanding whether a previous exposure to chronic stress may influence the prognosis of GBM.

## Results

### Corticosterone Administration Does Not Affect Overall Survival of a Syngeneic Mouse GBM Model

**Figure 1:**
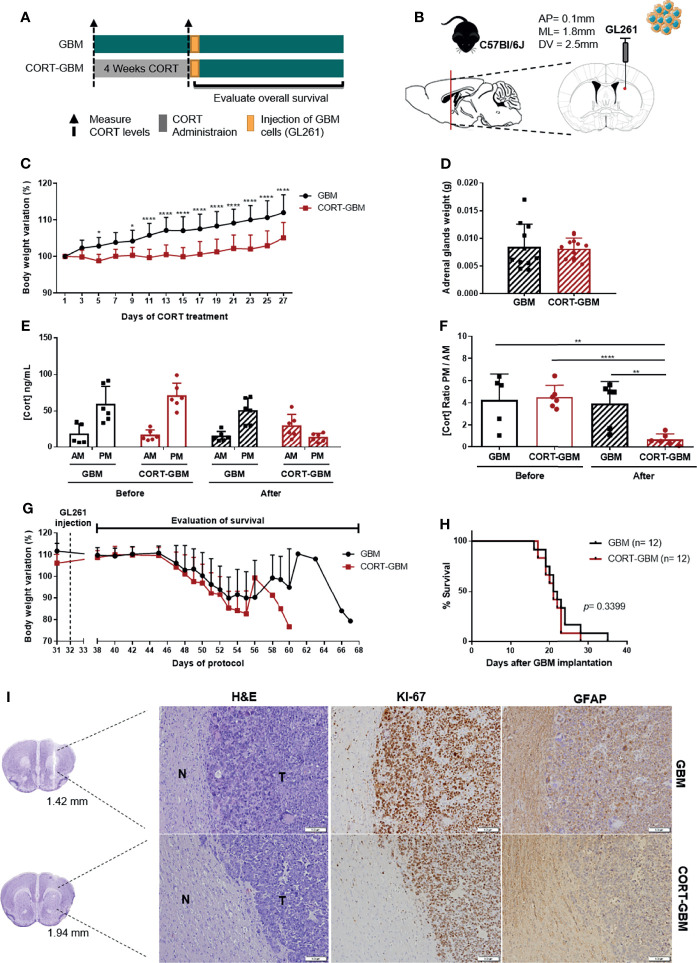
Corticosterone administration does not affect the survival of C57BL/6J mice orthotopically implanted with a mouse GBM cell line. **(A)** Experimental Design. C57BL/6J mice were subcutaneously injected daily with CORT (CORT-GBM group) or with the vehicle (GBM group) for 4 weeks before the orthotopic injection of a mouse GBM cell line, GL261, followed by the evaluation of mice body weight and overall survival. **(B)** Sagittal and coronal representation of the stereotactic injection site of GL261 cells into the mouse brain with the following coordinates from bregma (AP = 0.1 mm; ML = 1.8 mm; DV = 2.5 mm). **(C)** Body weight variation percentage from initial weight of GBM (black; n = 12) and CORT-GBM (red; n = 12) groups during administration of CORT, with multiple comparisons in each day. **(D)** Adrenal glands weight upon sacrifice of animals (n = 12 per group). **(E)** Serum CORT levels of GBM and CORT-GBM groups from morning (AM) and evening (PM) blood collections before and after the CORT administration (n = 6 per group). **(F)** Ratio of evening (PM) per morning (AM) CORT circulating levels of GBM and CORT-GBM groups before and after CORT administration (n = 6 per group). **(G)** Body weight variation percentage from initial weight of GBM (black; n = 12) and CORT-GBM (red; n = 12) groups after orthotopic GL261 injection. **(H)** Kaplan–Meier survival curves of GBM (black, n = 12) and CORT-GBM (red, n = 12) groups. **(I)** Representative coronal sections of the mouse brain area (GBM at 1.42 mm and CORT-GBM at 1.94 mm from bregma). Hematoxylin-eosin staining of mice brains presenting a tumor area (T) and surrounding non-tumor brain tissue (N). Immunohistochemical staining for Ki67 and GFAP of mice brains orthotopically-injected with GL261 cells of GBM group and CORT-GBM group. Magnification of 100x (Scale = 100 μm). *p < 0.05; **p < 0.01; ****p < 0.0001.

Chronic stress activates the HPA axis, leading to the secretion of GCs from the adrenal glands, namely corticosterone (CORT). CORT is an important effector of stress response, inducing diverse genomic and non-genomic effects in most cells of the organism. Importantly, GC signaling has been suggested as a putative pathway through which chronic stress can impact tumor progression (3). With this in mind, we decided to firstly use a paradigm of chronic exogenous CORT administration in a mouse model of GBM and determine the prognostic impact of hypercortisolemia in animals’ OS. C57BL/6J male mice were subjected to 4 weeks of subcutaneous injections of 20 mg/kg CORT (CORT-GBM group), while the control group was subcutaneously injected with vehicle (GBM group), before the orthotopic implantation of the GL261 mouse GBM cell line (**Figures 1A, B**).

This chronic exogenous CORT administration paradigm has been described to induce abrogated weight gain, and dysregulation of the HPA axis (27–29). To confirm the efficacy of this paradigm, animals’ body weight and adrenal glands weight upon animal’s sacrifice were recorded, and CORT circulating levels were measured at the beginning and at the end of the CORT administration protocol.

CORT administration significantly decreased mice body weight from day 9 until the end of the protocol (**Figure 1C**; 2-way ANOVA, F_(13, 286)_ = 11.50, *p* < 0.0001). Animals exposed to a chronic CORT administration (CORT-GBM) at sacrifice did not present significant differences in their adrenal glands weight as compared to control animals (GBM; **Figure 1D**). After the full protocol of CORT administration, there was an increase of CORT levels at the ante meridiem (AM) measurement and a decrease at the post meridiem (PM) measurement of the CORT-GBM group (**Figure 1E**). This reflects a dysregulation of the HPA axis in the CORT-GBM group with a significant decrease of the CORT ratio PM/AM (**Figure 1F**; t_10 =_ 8.015, *p* < 0.0001).

After GL261 orthotopic injection, the two groups were able to recover their weight from surgery, until the appearance of GBM-related symptoms and consequent weight loss (**Figure 1G**). No significant differences were found regarding the survival of CORT-GBM and GBM groups (**Figure 1H**; log rank test, *p* = 0.3399).

Chronic stress has also been suggested to affect cell proliferation and differentiation (1). Thus, we evaluated the expression pattern of Ki67, a proliferation marker commonly overexpressed in GBM, and GFAP, an astrocytic marker, using immunohistochemistry analyses. GL261-derived tumor cells stained positively for Ki67, while the major presence of GFAP positive cells was found in the periphery of the tumor, which is suggestive of astrogliosis. No major differences between CORT-GBM and GBM groups were found regarding the qualitative expression of these two proteins (**Figure 1I**).

### Previous Exposure to Chronic Restraint Stress Does Not Affect Overall Survival of a Human GBM Mouse Xenograft Model

**Figure 2:**
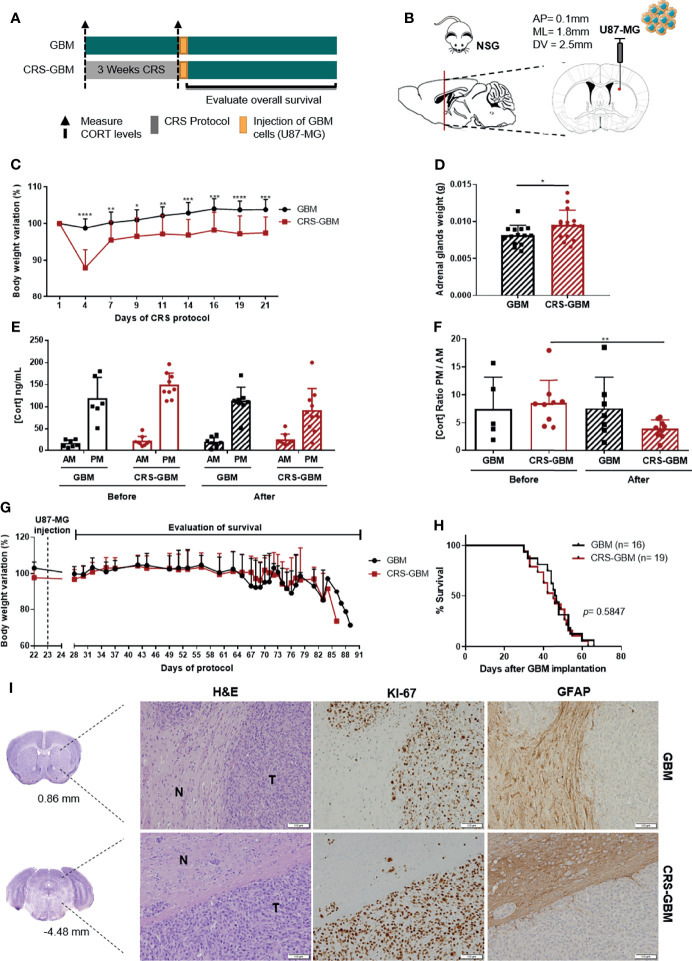
Chronic restraint stress does not affect overall survival in an immunocompromised xenograft model of GBM. **(A)** Experimental Design. NSG mice were subjected to 3 weeks of CRS (CRS-GBM group) or regular handling (GBM group) before the orthotopic injection of a human GBM cell line, U87-MG, followed by the evaluation of mice body weight and overall survival. **(B)** Sagittal and coronal representation of the stereotactic injection site of U87-MG cells into the mouse brain with the following coordinates from bregma (AP = 0.1 mm; ML = 1.8 mm; DV = 2.5 mm). **(C)** Body weight variation percentage from initial weight of GBM (black; n = 13) and CRS-GBM (red; n = 14) groups during CRS protocol representative of the two independent experiments. **(D)** Adrenal glands weight upon sacrifice of animals (GBM, n = 16; CRS-GBM, n = 19). **(E)** Serum CORT levels of GBM and CRS-GBM groups from morning (AM) and evening (PM) blood collections before and after the CRS protocol (GBM, n = 7; CRS-GBM, n = 9). **(F)** Ratio of evening (PM) per morning (AM) CORT circulating levels of GBM and CRS-GBM groups before and after the CRS protocol (GBM, n = 7; CRS-GBM, n = 9). **(G)** Body weight variation percentage from initial weight of GBM (black; n = 13) and CRS-GBM (red; n = 14) group after orthotopic injection of U87-MG GBM cells representative of the two independent experiments. **(H)** Kaplan–Meier survival curves of GBM (black, n = 16) and CRS-GBM (red, n = 19) groups in U87-MG glioma-bearing mice. **(I)** Representative coronal sections of the mouse brain area (GBM at 0.86 mm and CRS-GBM at -4.48 mm from bregma). Hematoxylin-eosin staining of mice brains presenting a tumor area (T) and surrounding non-tumor brain tissue (N). Immunohistochemical staining for Ki67 and GFAP of mice brains orthotopically-injected with U87-MG cells of GBM group and CRS-GBM group. Magnification of 100x (Scale= 100 μm). *p < 0.05; **p < 0.01; ***p < 0.001; ****p < 0.0001. Data from two independent *in vivo* experiments.

Elevated GC levels only represent a part of the stress response, not replicating all the neuroendocrine, psychological and physical changes. For that reason, the use of an established stress paradigm that recapitulates all dimensions of the stress response may be more appropriate. To understand if chronic stress could impact GBM aggressiveness, we studied an *in vivo* model using a human GBM cell line, which more closely mimics the human disease. Since most studies associating chronic stress with cancer progression in animal models are based on the restraint stress paradigm with immunocompromised mice (1, 2, 5, 30), we used a similar approach. NOD *scid* gamma (NSG) mice were exposed to a chronic restraint stress (CRS) or control protocol for 3 weeks before the orthotopic implantation of the human GBM cell line U87-MG (**Figures 2A, B**).

This CRS paradigm has been associated with abrogated weight gain and/or weight loss, increased adrenal glands weight, and to induce a dysregulation of HPA axis (1, 30, 31). We recorded animals’ body weight along the protocol, adrenal glands weights upon animals’ sacrifice, and measured CORT circulating levels at the beginning and at the end of the CRS protocol to control the efficacy of this paradigm. CRS had a significant impact in mice body weight variation from day 4 until the end of the protocol (**Figure 2C**; 2-way ANOVA, F_(8, 200)_ = 12.44, *p* < 0.0001). As expected, the adrenal glands of mice exposed to the CRS protocol were significantly heavier than control animals (**Figure 2D**; t_28_ = 2.263, *p* = 0.0316). The CRS protocol also led to a disruption of the HPA axis with a significant decrease of the CORT ratio PM/AM of the CRS-GBM group (**Figure 2F**; t_16_ = 3.136, *p* = 0.0064). All of these measures indicate that the stress protocol was effective.

After implantation of U87-MG cells, both groups recovered their body weight, until the appearance of GBM-related symptoms and consequent weight loss (**Figure 2G**). No significant differences regarding the OS of CRS-GBM and GBM groups were found (**Figure 2H**; log rank test, *p* = 0.5847). U87-MG-derived tumor cells stained positively for Ki67, but the majority of the tumor cells were negative for GFAP (**Figure 2I**). The periphery of the tumor (reactive border) presented a strong staining for GFAP (**Figure 2I**). This pattern of expression was similar between both control and CRS groups.

### Previous Exposure to Chronic Unpredictable Stress Does Not Affect Overall Survival of a Mouse GBM Model

The stress response is known to produce remarkable changes in the immune system, which can compromise cellular immunity and, thus, contribute to facilitate tumor initiation, progression and aggressiveness (32). Both SNS system and HPA axis mediators regulate distinct aspects of immune function, including antigen presentation, T cell proliferation, and cell-mediated and humoral immunity (3, 32). Thus, using an immunocompetent GBM mouse model with an aggressive stress paradigm was mandatory to evaluate a possible effect in immunomodulation that could impact GBM.

**Figure 3:**
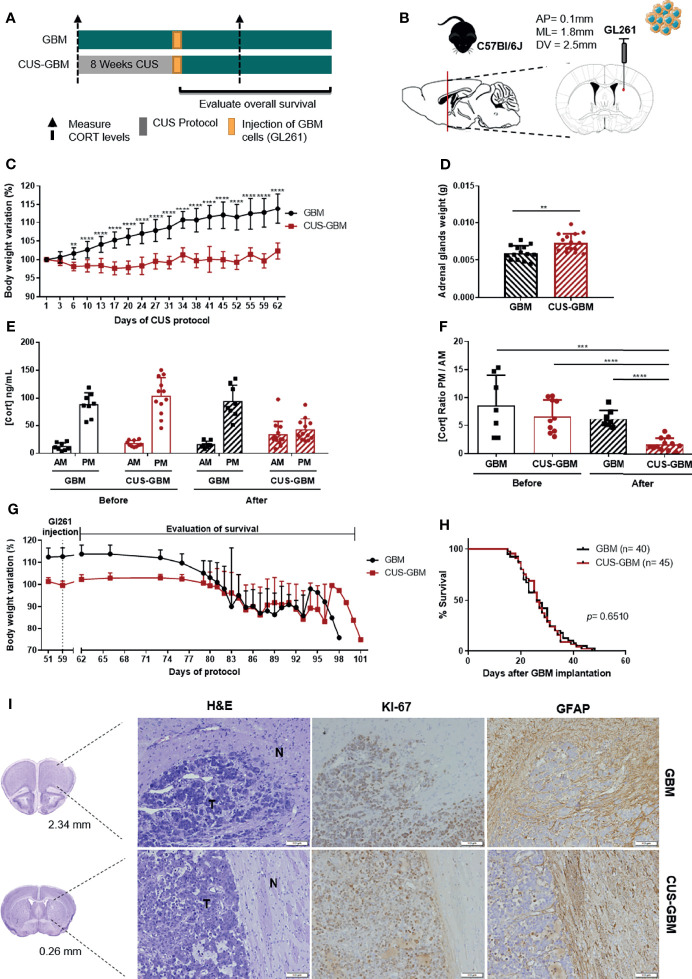
Chronic unpredictable stress does not affect overall survival of an immunocompetent mouse GBM model. **(A)** Experimental Design. C57BL/6J mice were subjected to 8 weeks of CUS before (CUS-GBM group) the orthotopic injection of a mouse GBM cell line, GL261; the control group was subjected to regular handling (GBM group). **(B)** Sagittal and coronal representation of the stereotactic injection site of GL261 cells into the mouse brain with the following coordinates from bregma (AP = 0.1 mm; ML = 1.8 mm; DV = 2.5mm). **(C)** Body weight variation percentage from initial weight of GBM (black; n = 15), and CUS-GBM (red; n = 15) groups during the protocol of CUS before GBM implantation representative of the four independent experiments. **(D)** Adrenal glands weight upon sacrifice of animals (GBM, n = 14; CUS-GBM, n = 15). **(E)** Serum CORT levels of GBM and CUS-GBM groups from morning (AM) and evening (PM) blood collections before and after the CUS protocol (GBM, n = 8; CUS-GBM, n = 12). **(F)** Ratio of evening (PM) per morning (AM) CORT circulating levels of GBM and CUS-GBM groups before and after the CUS protocol (GBM, n = 8; CUS-GBM, n = 12). **(G)** Body weight variation percentage from initial weight of GBM (black; n = 15), and CUS-GBM (red; n = 15) groups after GBM implantation representative of the four independent experiments. **(H)** Kaplan–Meier survival curves of GBM (black, n = 40), and CUS-GBM (red, n = 45) groups. **(I)** Representative coronal sections of the mouse brain area (GBM at 2.34 mm and CUS-GBM at 0.26 mm). Hematoxylin-eosin staining of mice brains presenting a tumor area (T) and surrounding non-tumor brain tissue (N). Immunohistochemical staining for Ki67 and GFAP of mice brains orthotopically-injected with GL261 cells of GBM and CUS-GBM groups. Magnification of 100x (Scale = 100 μm). **p < 0.01; ***p < 0.001; ****p < 0.0001. Data from 4 independent *in vivo* experiments.

The chronic unpredictable stress (CUS) protocol is commonly used to study the impact of stress in animal models and involves the daily exposure to a variety of stressors that are presented in a random, intermittent, and unpredictable form during several weeks. The variety of stressors includes social defeat, restraint, overcrowding, hot drier, shaking, inverted light cycle and overnight illumination. C57BL/6J mice were subjected to a CUS protocol for 8 weeks (CUS-GBM group) before orthotopic injection of a mouse GBM cell line (GL261) (**Figures 3A, B**). This CUS protocol has been described to induce abrogated weight gain, and to induce alterations in the adrenal glands, because they continuously stimulate the synthesis of stress hormones that can lead to morphological alterations (33, 34). Moreover, previous works have suggested a higher adrenal glands weight after a stress protocol in mice (33, 35, 36). Therefore, to confirm the efficacy of the CUS protocol, animals’ body weight was recorded, and for the GBM and CUS-GBM groups we determined the adrenal glands weight upon animals’ sacrifice and the CORT circulating levels at the beginning and at the end of the CUS protocol.

CUS significantly decreased mice body weight of the CUS-GBM group when compared to the GBM group from day 6 until the end of the CUS protocol (**Figure 3C**; 2-way ANOVA, F_(17,504)_ = 18.64, *p* < 0.0001). Animals exposed to a CUS protocol before the orthotopic implantation of GBM presented a statistically significant increase in adrenal glands weight (**Figure 3D**; t_27_ = 3.319, *p* = 0.0026), suggesting an effective stress protocol. After the CUS protocol, there was a dysregulation of the HPA axis in the CUS-GBM group, with a significant decrease of the CORT ratio PM/AM (**Figure 3F**; t_20_ = 5.449, *p* < 0.0001). At later timepoints, coincident with the appearance of GBM-related symptoms, significant weight loss was observed for all groups (**Figure 3G**). No significant differences regarding OS were found (**Figure 3H**; log rank test, *p* = 0.8026). The immunohistochemistry for Ki67 and GFAP proteins did not reveal major differences between groups (**Figure 3I**).

**Figure 4:**
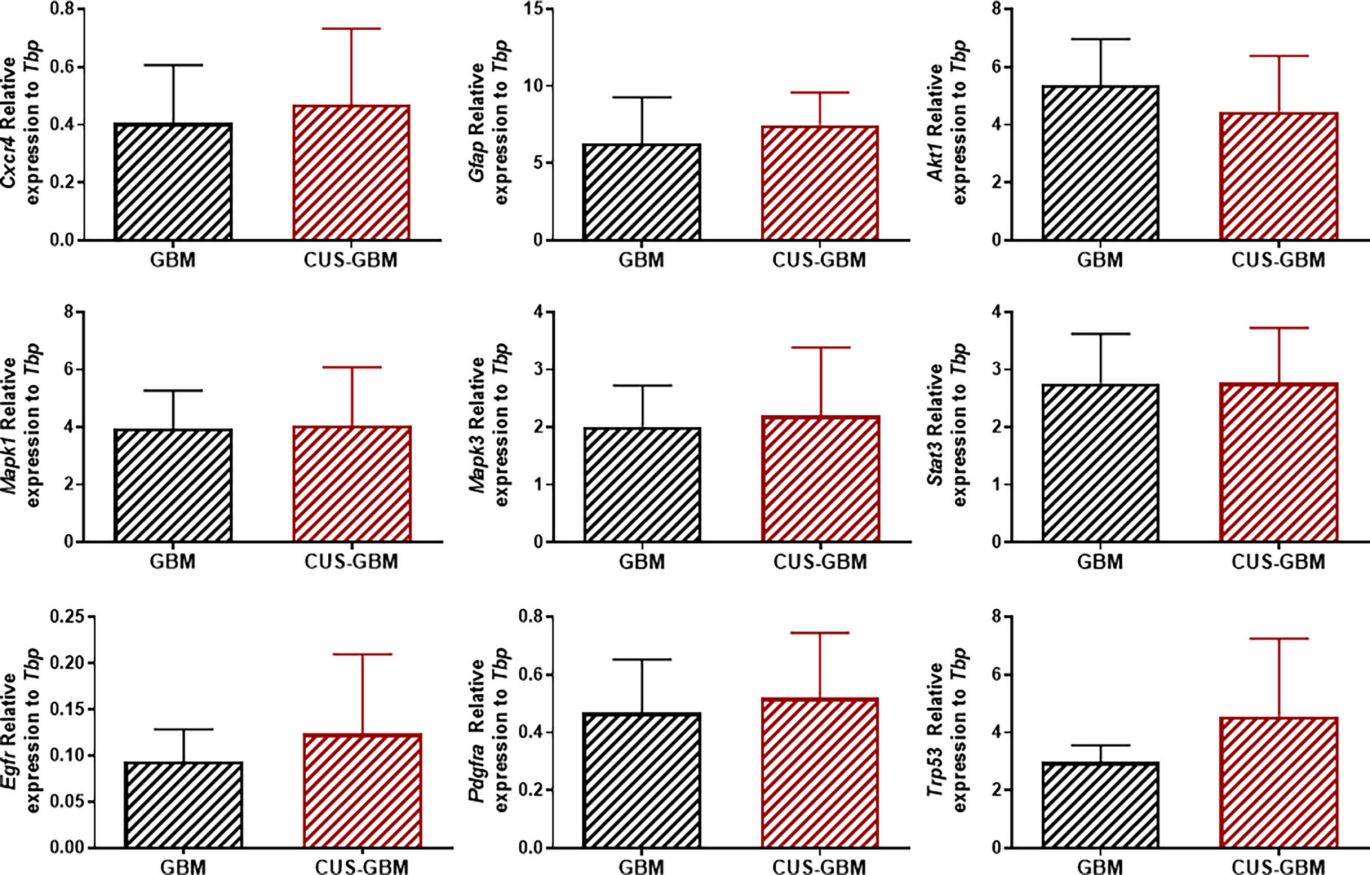
Chronic stress does not affect the expression of several genes in tumor tissue from mice implanted with GBM cells. Quantification of *Cxcr4, Gfap, Akt1, Mapk1, Mapk3, Stat3, Egfr, Pdgfra* and *Trp53* genes expression in mice unexposed or exposed to CUS protocol in tumor tissues (expression normalized to *Tbp*). (GBM, n = 3; CUS-GBM n = 5).

To further understand if the CUS protocol could affect GBM aggressiveness at the molecular level, we performed qRT-PCR analyses in *ex vivo* tumor tissues collected from these mice for genes associated with GBM aggressiveness, such as *Cxcr4*, *Gfap, Akt1*, *Mapk1*, *Mapk3*, *Stat3*, *Egfr*, *Pdgfra* and *Trp53*. No significant differences were found in gene expression levels between each experimental group (**Figure 4**; unpaired *t*-test).

## Discussion

GBM accounts for 80% of malignant primary brain tumors in adults, and remains the most lethal, with a median OS of 14.6 months after diagnosis (18, 37). Despite considerable progress in the understanding of the biological characteristics of GBM, this cancer is still associated with very poor prognosis (18, 38). The complete understanding of the involvement of bio-behavioral factors in cancer is relevant towards preventive measures and for the awareness of risk factors that can impact patients’ prognosis.

Evidences from animal and human studies suggest the implication of chronic stress in the aggressiveness of cancer (5, 39–42). Nevertheless, the association between psychological stress and cancer remains enigmatic, with some possible biological mechanisms, such as dysregulation of the neuroendocrine axis and impairment of immune functions, being proposed and linked with some cancer types (3, 23, 43, 44). There are multiple biological mechanisms that underlie the link between stress and cancer, and, as a result, the effects of stress may vary across cancer types (3).

Evidence of the influence of bio-behavioral factors on GBM has been previously documented. Previous exposure to environmental enrichment in mice before orthotopic implantation of a mouse GBM cell line leads to a prolonged survival and reduced glioma growth (45). This is evidence that the brain microenvironment can be modulated by environmental factors, such as prolonged sensory, social and physical experiences, ultimately influencing the aggressiveness of brain cancer (45). In fact, some paracrine interactions between glioma cells and the brain microenvironment have been indicated to influence glioma pathophysiology, as well as microglial cells contributing for GBM cell invasion and non-neoplastic astrocytes being able to convert into a reactive phenotype by the glioma microenvironment (46, 47). Mechanistic investigations have documented a possible mechanism through which the tumor microenvironment modulated GBM pathophysiology, where they found a crosstalk between GBM and glial cells (48). The reward system can also manipulate tumor growth. A recent study was able to establish a causal link between brain’s reward system manipulation and tumor growth that is dependent on SNS activity, with an anti-tumor immune response (8). These findings elucidate how positive stimuli and patient’s psychological state can impact cancer progression. Still, the impact of GCs and/or chronic stress in GBM remains uncertain.

Our study provides novel insights on the putative effects of a pre-exposure to chronic stress in GBM aggressiveness. We demonstrated through *in vivo* approaches with mouse GBM models that CORT and chronic stress, both in immunocompetent and immunodeficient contexts, do not affect GBM prognosis and aggressiveness.

Chronic stress results in systemic elevated levels of catecholaminergic neurotransmitters and GCs that are able to regulate cellular processes such as inflammation, apoptosis and cellular immune response (49, 50). Previous studies suggested that chronic stress can contribute to increased tumor growth through GCs signaling, since they regulate a wide variety of cellular processes and physiologic functions through genomic and non-genomic actions (50, 51). A study with clinical and mouse experimental data suggested that dexamethasone, a synthetic GC with potent anti-inflammatory activity, may decrease the effectiveness of treatments and shorten survival in GBM patients (52). Furthermore, dexamethasone treatment of human GBM primary cells fostered a glioma stem cell-like phenotype, typically associated with more aggressive and malignant features (53). Our findings suggest that exogenous administered CORT does not affect the OS of a mouse GBM model. This is of interest, because GCs have been described to present different roles in cancer (54). For example, dexamethasone induced proliferation of tumor cells in a pre-clinical lung carcinoma mouse model (55). On the other hand, low-dose of dexamethasone suppressed ovarian cancer progression and metastasis in an immunocompetent syngeneic mouse model (56). It is important to refer that this protocol only mimics part of the stress response, not completely replicating all the physiological changes induced by stress. In this perspective, it has been described that both catecholamines and GCs can act in a synergistic fashion to facilitate cancer growth (3, 20, 50). For example, cortisol increased beta-adrenergic receptors density with increased cAMP accumulation in lung carcinoma cells (57). Interestingly, previous studies suggested the existence of direct effects of beta-adrenergic signaling in models of GBM, particularly in *in vitro* contexts, where both propranolol and isoproterenol suppressed the proliferation of human glioblastoma cell lines (58), and treatment of cancer cells with propranolol counteracts the epidermal growth factor receptor (EGFR) oncogenic traits (59), that is associated with GBM aggressiveness features. So, it is plausible that a chronic stress paradigm that mimics more closely the stress response with the increase of both catecholamines and GC levels may lead to higher impact on cancer (44). Also, the effects of CORT injection are time-dependent, and as soon as the last injection is administered, the cumulative effective can start to be lost. Therefore, future studies are warranted to properly address the impact of GC and adrenergic signaling in different tumor types *in vivo*.

A wide variety of stress paradigms with animal models have been used to study the causal effect of stress in cancer aggressiveness. The majority of these studies used xenograft cancer models with a CRS protocol (1, 2, 5). We demonstrated that a previous exposure to a CRS protocol did not impact GBM aggressiveness in an immunocompromised xenograft model of GBM. Conversely, the CRS paradigm has been shown to promote colorectal cancer growth in a xenograft mouse model (1). However, it has also been reported that restraint stress alone did not significantly promote colorectal cancer growth in a similar xenograft mouse model (60). Another study showed that CRS did not decrease the survival of an oral squamous cell carcinoma mouse model (61).

The stress response is also known to produce remarkable changes in the immune system, which can compromise cellular immunity with down-regulation of the cellular immune response (3, 32, 44, 62). Malignant tumors also develop multiple escape mechanisms through which they evade recognition and destruction by the immune system (63). Considering that NSG mice are severely immunocompromised, which could affect tumor aggressiveness and interfere with survival, we also explored immunocompetent models, in which we could account for the contribution of the immune system in tumor progression. Therefore, the use of a paradigm that comprised all physiological parameters of the stress response is of extreme importance. The CUS protocol is of long-term duration and commonly used to study the impact of stress in animal models and is characterized by the random, intermittent, and unpredictable exposure to a variety of different stressors ultimately leading to a more aggressive phenotype (33, 34). Mice exposed to the CUS protocol before GBM implantation did not present any significant differences in OS. This suggests that, in this GBM model, previous exposure to chronic stress does not affect tumor aggressiveness.

Consistent with our survival results, histological and molecular analyses did not show any significant difference between groups of the different stress paradigms that we tested. It would be expected that a more aggressive GBM phenotype would present increased proliferation activity or increased expression of some genes related to GBM aggressiveness. We should denote that GBM is a highly heterogeneous cancer, and we can have different degrees of aggressive phenotypes, as observed between individuals from the same group. Also, since these samples were obtained at the final endpoint of mice survival, this could affect the comparison between animals as each was collected at different time-points. Since the tumors were all at the same final stage, independently of the time they take to reach it, an established fixed time-point for sacrificing animals could address this question in future studies. However, the outcome of survival is of the utmost importance to answer this hypothesis, and not always an increased expression of proliferation markers or tumor size can predict the outcome.

Our findings were surprising in the light of other studies suggesting that stress/GCs can impact cancer initiation and progression (1–3, 5, 6, 42). Though several factors could influence the outcome of these experiments, it is important to refer that stress may impact differently in very distinct cancer types, and the GBM models we used in this work are very aggressive and of fast progression, leading to a short OS that may limit the temporal window to observe a putative impact of stress, particularly if that effect is not very pronounced. Nonetheless, these validated mice models recapitulate the extremely malignant behavior and clinical presentation of GBM, one of the most aggressive human cancers, with patients presenting an extremely poor survival. In addition, we used 1 mouse and 1 human GBM cell lines, so we need to have in consideration the specificity of each model and that GBM is highly heterogeneous. On the other hand, different strains have different susceptibilities to stress (64–66). In this perspective, less aggressive GBM models could be interesting to study in order to complement these findings. For example, one could use a genetic model where there is already a predisposition for GBM formation (67). For example, the Cre/Lox mouse model hGFAP-Cre^+^;p53^lox/lox^;Pten^lox/+^ of glioma which results in 73% of mice developing grade III and grade IV gliomas at a median latency of seven months (68); the RCAS/Ntv-a mouse model Chk2^+/−^ of glioma which presents an average survival of 55 days, and with 40% of mice developing grade IV gliomas (69). These models would be very interesting to identify the effects of stress on GBM initiation, degree of malignancy, penetrance, and survival.

In this study, we provide evidence regarding the prognostic impact of a previous exposure to chronic stress and GCs in GBM. By using *in vivo* approaches, we demonstrate that prolonged pre-exposure to chronic stress/GCs does not impact mice OS, both in the context of a human and a mouse GBM cell line model. Nonetheless, additional studies are needed using other models to fully exclude a putative contribution of stress for GBM pathophysiology, at different stages and dimensions of the disease, including tumor initiation, progression, and aggressiveness.

## Material and Methods

### Animals

Ten weeks old male C57BL/6J mice were obtained from Charles River Laboratories (027), and female NOD.Cg-*Prkdc^scid^ Il2rg^tm1Wjl^*/SzJ (NOD *scid* gamma, NSG) mice were obtained from Charles River Laboratories (005557). Mice were housed 4-5 per cage under standard environmental conditions, light/dark cycle of 12/12 hours with lights on at 8 AM; 22°C of room temperature (RT) and a relative humidity of 55%; with *ad libitum* access to food and water. Animals were handled twice per day for 2 weeks before the experiments. CD-1 IGS male mice used in the CUS protocol were purchased with 12-weeks-old from Charles River (022) and housed individually under the same conditions. All experiments were performed in agreement with the European Union Directive 2010/63/EU, and approved by the national ethical committee DGAV (Direção Geral de Alimentação e Veterinária, reference no. 008516). Sentinel mice housed in the same room were used to confirm the specified pathogen-free health status of the mice as recommended by the FELASA guidelines.

### Cell Culture

The established human GBM U87-MG cell line (kindly provided by Dr. Joseph Costello, University of California, San Francisco) and the mouse GBM GL261 cell line (kindly provided by Dr. Maria Conceição de Lima, University of Coimbra) were used in this study. Cells were cultured in Dulbecco’s Modified Eagle Medium (DMEM; Biochrom GmbH, Berlin, Germany) supplemented with 10% Fetal Bovine Serum (FBS; Biochrom GmbH, Berlin, Germany) and maintained in a humidified atmosphere at 37°C and 5% of CO_2_. For the *in vivo* orthotopic injections, GBM cells were trypsinized and viable cells were counted using Trypan Blue (Gibco) in a Neubauer chamber. Cell suspension was centrifuged and resuspended in the proper volume of cold phosphate buffered saline (PBS 1x) for further orthotopic injection (5 µL/animal).

### Intracranial Surgery and *In Vivo* Assays

For the orthotopic injection of GBM cells animals were anesthetized with a mixture of ketamine (Imalgene, Merial, USA; 75 mg/kg) and medetomidine hydrochloride (Dorbene, Zoetis, Spain; 1 mg/kg) intraperitoneally injected), and analgesia was achieved with butorphanol (5 mg/kg, subcutaneously injected). Mice were placed on a stereotaxic head frame (Stoelting, USA) and a small incision in the skin was made and a burr hole was drilled in the skull. 2x10^4^ GL261 or 2x10^5^ U87-MG cells were injected using a point style 4 beveled 26s-gauge needle 10 µL Hamilton syringe at 1.7 µL/min in the right striatum (1.8 mm mediolateral, 0.1 mm anteroposterior, and 2.5 mm dorsoventral from the bregma). After injection, the needle was left in place for 2 min to avoid any backflow from the needle tract. Mice body weight was measured regularly to assess stress efficacy and later tumor-related symptoms, and the behavior and symptomatology was monitored daily. For the evaluation of OS, humane endpoints for sacrifice were used when any of the following conditions was observed: severe weight loss (> 30% of maximum body weight), and moribund condition. Mice were sacrificed with a lethal dose of anesthesia injected intraperitoneally. Animals assigned for histological analysis were perfused with saline solution followed by 4% paraformaldehyde (PFA) and brains were collected immediately and stored in 4% PFA until embedding in paraffin. Animals assigned for molecular analysis were decapitated after anesthesia overdose and the head was immersed for 5 s in liquid nitrogen (snap-freeze technique) followed by macrodissection of tumor tissue. Adrenal glands were collected and weighed in an analytical balance immediately after sacrifice.

### Stress Protocols

CORT Administration: The chronic CORT administration protocol consists in daily subcutaneous injections of CORT for 4 weeks at 20 mg/kg in 1% ethanol and delivered with sesame oil as vehicle (28, 29, 31). The efficacy of the stress protocols was confirmed by body weight alterations, adrenal glands weight measurements, and CORT circulating levels determination. CRS Protocol: The CRS protocol consists of 3 weeks of restraint of mice for 2 h in the morning in 50 mL plastic tube (Falcon) with holes, as previously described (1, 35). CUS Protocol: The CUS protocol consists of 8 weeks of daily exposure to several different stressors presented in a random order and in an unpredictable form. The different types of stressors are: shaking – groups of 4/5 mice are placed in a plastic box container and placed in an orbital shaker for 2 h at 150 rpm; overcrowding – groups of 8/9 mice are placed in a plastic box container for 3 h; restraint - mouse is placed in a 50 mL plastic tube (Falcon) with openings in the front and sides to allow the breathing of animals, for 3 h; hot drier – mice are exposed to a hot airstream from a hair dryer for 15 min; social defeat – mice are introduced in a cage of an aggressive mice (CD-1 IGS Mouse) and after being defeated, they are placed in a transparent and perforated plastic container to avoid further physical contact, inside the resident home cage for 5-20 min; overnight illumination – mice are exposed to regular room light during the night period; and inverted light cycle – regular room light is off during day time and on during night time for 2 days (33).

### Blood Collection and Serum CORT Analysis

For measuring circulating CORT levels, tail blood was collected in a subset of animals before and after the stress paradigms (2 collections were performed – morning (8 AM) and evening (8 PM). Collections were made in less than 2 min after taking the animal from its homecage. After collection, the blood was centrifuged for 10 min at 13 000 xg and serum (supernatant) was stored at -80°C until analysis. Serum CORT concentration was determined using a commercially available immunoassay kit (DetectX Corticosterone Enzyme Immunoassay Kit, Arbor Assays, Ann Arbor, MI, USA; #K014-H5) according to the manufacturer’s instructions. Assay sensitivity was 18.6 pg/mL.

### Immunohistochemistry

Tissues were formalin-fixed and paraffin-embedded, and cut in 4 μm slices. Paraffin wax was removed, and the sample rehydrated in an autostainer (Leica XL) by immersing the slides in a sequence of xylene, ethanol absolute, ethanol 96%, ethanol 70% and water. Before the antigen retrieval, the Ki67 slides were washed with TBS-Tween 0.5% for 10 min followed with TBS 1x. Antigen retrieval was carried out using Heat Induced Epitope Retrieval (HIER), through the immersion of the slides in a Sodium Citrate Buffer (10 mM Sodium Citrate, 0.05% tween 20, pH 6.0), for 20 min. Then slides were incubated in 3% hydrogen peroxide (H_2_O_2_) for 10 min. The UltraVision Large Volume Detection System Anti-Polyvalent HRP (LabVision Corporation, Thermo Scientific) was used. The blocking solution (LabVision kit) was applied for 30 min, then the respective primary antibody was applied, Ki67 (#550609, BD Bioscience, 1:200) and GFAP (#Z0334, DAKO, 1:2000) diluted in the LabVision kit Primary Ab diluent, and incubated overnight at 4°C. A biotinylated goat secondary antibody (LabVision kit) was applied followed by the streptavidin peroxidase (LabVision kit) and 3,3’-diaminobenzidine (DAB) substrate used as chromogen (1 mL of DAB substrate buffer + 1 drop of DAB chromogen, DAKO). After rinse in TBS 1x and in running water, the contrast and counterstain was performed in the autostainer (Leica XL) by immersing the slides in a sequence of running water, Harris Hematoxylin (25% for Ki67 and 50% for GFAP), running water, ammoniacal water 0.5%, running water, ethanol 96%, ethanol absolute and Xylol. Slides were mounted using entellan. The immunohistochemistry photos were taken with an Olympus BX61 microscope using the CellSens Dimension software at 100x magnification.

### Quantitative Reverse Transcriptase-Polymerase Chain Reaction (qRT-PCR)

Total RNA from tumor tissue (collected when animals were sacrificed) was extracted using *Trizol Reagent* from Invitrogen. One µg of total RNA (quantified by a nanodrop Spectophotometer ND-1000) was reverse transcribed into complementary DNA (cDNA) using High Capacity cDNA Reverse Transcription Kit from Applied Biosystems.

The expression levels of mouse mRNA transcripts C-X-C motif chemokine receptor 4 (Cxcr4, GeneID: 12767), glial fibrillary acidic protein (Gfap, GeneID: 14580), AKT serine/threonine kinase 1 (Akt1, GeneID: 11651), mitogen-activated protein kinase 1 (Mapk1, GeneID: 26413), mitogen-activated protein kinase 3 (Mapk3, GeneID: 26417), signal transducer and activator of transcription 3 (Stat3, GeneID: 20848), epidermal growth factor receptor (Egfr, GeneID: 13649), platelet derived growth factor receptor alpha (Pdgfra, GeneID: 18595), and transformation related protein 53 (Trp53, GeneID: 22059) were assessed by qRT-PCR assays. The TATA-binding protein, (Tbp, GeneID: 21374) was used as reference gene. Primer set sequences are detailed in **Table S1**. A kit from KAPA SYBR^®^ FAST qPCR Master Mix (2X) Universal was used. The reactions were performed in duplicate and run on a Thermal cycler CFX96 using the program Bio-Rad CFX Manager. The conditions of PCR were as follows: 3 min at 95°C; followed by 40 cycles of denaturation: 3 s at 95°C, 30 s at respective melting temperature for annealing (Tm; **Table S1**) and 30 s at 72°C for extension; the dissociation was performed by 5 s at 65 °C with increasing the temperature in 1°C from 65°C to 95°C. PCR products weight were confirmed on 2% agarose gels. Gene expression was evaluated by relative quantification using the delta Ct method (ΔCt) and each gene was normalized to the reference housekeeping TBP gene.

### Statistical Analysis

Statistical analysis was performed using IBM SPSS Statistics version 24 and graph’s representation using Graph-Pad Prism version 6. To determine statistical differences between groups in the adrenal glands weight and in CORT Ratio PM/AM, two-sided unpaired *t*-test was applied. Analysis of the overall survival was performed using the log-rank test. Analysis of body weight variance between groups was performed using two-way analysis of variance (ANOVA) followed by the *post-hoc* Bonferroni test for multiple comparisons. The results are expressed as group means ± SD (standard deviation) and the level of significance in all the statistical analysis was set at *p* < 0.05.

## Data Availability Statement

The raw data supporting the conclusions of this article will be made available by the authors, without undue reservation.

## Ethics Statement

The animal study was reviewed and approved by Direção Geral de Alimentação e Veterinária.

## Author Contributions

Conceptualization, NS, AR, and BMC. Methodology, ML, JC, MP, CG, EM, BC, AR, and BMC. Software, NS, AR, and BMC. Validation, ML, AR, and BMC. Formal analysis, ML, AR, and BMC. Investigation, ML, JC, IS, NS, AR, and BMC. Resources, NS, IS, AR, and BMC. Data curation, ML, JC, IS, NS, AR, and BMC. Writing—original draft preparation: ML, AR, and BMC. Writing—review and editing: all authors. Visualization, ML, AR, and BMC. Supervision, AR and BMC. Project administration, NS, AR, and BMC. Funding acquisition, NS, IS, AR, and BMC. All authors have read and agreed to the published version of the manuscript.

## Funding

This research was funded by FEDER funds through the Operational Programme Competitiveness Factors–COMPETE and National Funds through FCT under the projects UIDB/50026/2020, UIDP/50026/2020, and POCI-01-0145-FEDER-007038; by the project NORTE-01-0145-FEDER-000013, NORTE-01-0246-FEDER-000012, and NORTE-01-0145-FEDER-000023, supported by Norte Portugal Regional Operational Programme (NORTE 2020), under the PORTUGAL 2020 Partnership Agreement, through the European Regional Development Fund (ERDF). JC, CG, EM, and BMC was funded by FCT-Foundation for Science and Technology (SFRH/BD/88121/2012 to JC; SFRH/BD/92786/2013 to CG; PD/BDE/143154/2019 to EM; and PTDC/SAUGMG/113795/2009, IF/00601/2012 and CEECIND/00072/2018 to BC). BC was also funded by Fundação Calouste Gulbenkian and Liga Portuguesa Contra o Cancro.

## Conflict of Interest

The authors declare that the research was conducted in the absence of any commercial or financial relationships that could be construed as a potential conflict of interest.

## Publisher’s Note

All claims expressed in this article are solely those of the authors and do not necessarily represent those of their affiliated organizations, or those of the publisher, the editors and the reviewers. Any product that may be evaluated in this article, or claim that may be made by its manufacturer, is not guaranteed or endorsed by the publisher.

## Supplementary Material

Supplementary Table 1 Sequence of primers used for qRT-PCR analyses and respective melting temperatures (Tm).
