# Supplementary material for: Chronic Stress Does Not Influence the Survival of Mouse Models of Glioblastoma
Source: Front Oncol. 2022 Mar 25;12:856210. doi: 10.3389/fonc.2022.856210 (PMC8990973; doi:10.3389/fonc.2022.856210)
Supplement: Supplementary Table 1 — Sequence of primers used for qRT-PCR analyses and respective melting temperatures (Tm). [file Table_1.docx]

Supplementary Material

**Table S1.** Sequence of primers used for qRT-PCR analyses and respective melting temperatures (Tm).

| Gene | Primer Forward | Primer Reverse | Tm °C |
| --- | --- | --- | --- |
| *Cxcr4* | 5’–ACGGCTGTAGAGCGAGTGTT–3’ | 5’–GTAGATGGTGGGCAGGAAGA–3’ | 60 |
| *Gfap* | 5’–CTTGACCTGCGATCTGGAGT–3’ | 5’–GTAGGTGGCGATCTCGATGT–3’ | 59 |
| *Tbp* | 5’–GGGAGAATCATGGACCAGAA–3’ | 5’–TTGCTGCTGCTGTCTTTGTT–3’ | 55 |
| *Stat3* | 5’–GAGCTGGCTGACTGGAAGAG–3’ | 5’–TTGTTGGCGGGTCTGAAGTT–3’ | 60 |
| *Akt1* | 5’–TCAGGGGCTGAAGAGATGGA–3’ | 5’–GCGACGATGACCTCCTTCTT–3’ | 60 |
| *Pdgfra* | 5’–GGGAGGACGTTCAAGACCAG–3’ | 5’–TAAAGACGGCACAGGTCACC–3’ | 60 |
| *Egfr* | 5’–AGACCACTTTCTGAGCCTGC–3’ | 5’–AGGGATTCTCTCCACGGTGT–3’ | 60 |
| *Trp53* | 5’–AACTATGGCTTCCACCTGGG–3’ | 5’–CTCCGTCATGTGCTGTGACT–3’ | 60 |
| *Mapk3* | 5’–TGGCTGAGATGCTCTCCAAC–3’ | 5’–CCTTGGTTTTCGAGGGCAGA–3’ | 60 |
| *Mapk1* | 5’–CCTTTTGAGCACCAGACCTACT–3’ | 5’–GCTTGTAAAGGTCCGTCTCCA–3’ | 60 |
